# The Relationship between Body Composition, Dietary Intake, Physical Activity, and Pulmonary Status in Adolescents and Adults with Cystic Fibrosis

**DOI:** 10.3390/nu14020310

**Published:** 2022-01-12

**Authors:** Kevin J. Scully, Laura T. Jay, Steven Freedman, Gregory S. Sawicki, Ahmet Uluer, Joel S. Finkelstein, Melissa S. Putman

**Affiliations:** 1 Division of Endocrinology, Boston Children’s Hospital, Boston, MA 02115, USA; kevin.scully@childrens.harvard.edu; 2 Harvard Medical School, Boston, MA 02115, USA; sfreedma@bidmc.harvard.edu (S.F.); gregory.sawicki@childrens.harvard.edu (G.S.S.); Ahmet.Uluer@childrens.harvard.edu (A.U.); finkelstein.joel@mgh.harvard.edu (J.S.F.); 3 Division of Gastroenterology, Hepatology and Nutrition, Boston Children’s Hospital, Boston, MA 02115, USA; laura.jay@childrens.harvard.edu; 4 Division of Gastroenterology, Beth Israel Deaconess Hospital, Boston, MA 02115, USA; 5 Division of Pulmonary Medicine, Boston Children’s Hospital, Boston, MA 02115, USA; 6 Division of Pulmonary and Critical Care Medicine, Brigham and Women’s Hospital, Boston, MA 02115, USA; 7 Division of Endocrinology, Massachusetts General Hospital, Boston, MA 02115, USA

**Keywords:** body composition, cystic fibrosis, dual-energy X-ray absorptiometry, lean body mass, appendicular lean mass index, fat mass index, dietary intake

## Abstract

Measures of body fat and lean mass may better predict important clinical outcomes in patients with cystic fibrosis (CF) than body mass index (BMI). Little is known about how diet quality and exercise may impact body composition in these patients. Dual X-ray absorptiometry (DXA) body composition, 24-h dietary recall, and physical activity were assessed in a cross-sectional analysis of 38 adolescents and adults with CF and 19 age-, race-, and gender-matched healthy volunteers. Compared with the healthy volunteers, participants with CF had a lower appendicular lean mass index (ALMI), despite no observed difference in BMI, and their diets consisted of higher glycemic index foods with a greater proportion of calories from fat and a lower proportion of calories from protein. In participants with CF, pulmonary function positively correlated with measures of lean mass, particularly ALMI, and negatively correlated with multiple measures of body fat after controlling for age, gender, and BMI. Higher physical activity levels were associated with greater ALMI and lower body fat. In conclusion, body composition measures, particularly ALMI, may better predict key clinical outcomes in individuals with CF than BMI. Future longitudinal studies analyzing the effect of dietary intake and exercise on body composition and CF-specific clinical outcomes are needed.

## 1. Introduction

Nutritional optimization has long been a focus of care in patients with cystic fibrosis (CF), with body mass index (BMI) being utilized as the primary marker of health and survival [1,2,3]. Undernutrition in CF has been associated with worsening pulmonary status, decreased exercise tolerance, immunologic impairment, impaired growth, decreased quality of life, and a shorter life expectancy [4,5,6]. Conversely, optimized nutritional status is associated with improved lung function, clinical outcomes and survival [7,8]. As a result of this, nutritional guidelines recommend maintaining a body mass index (BMI) at or above the 50th percentile of age for children and adolescents, and a level of at least 22 kg/m^2^ in adult females and 23 kg/m^2^ in adult males aged 18 and over. In the past, this concern for malnutrition has often led to physicians recommending high-calorie diets without concern for diet quality [9]. This has led to a tendency for individuals with CF to overconsume energy-dense, nutrient-poor foods, particularly foods high in added sugars and refined carbohydrates that have a high glycemic index [9,10,11,12].

Life expectancy and clinical outcomes for patients with CF have significantly improved with widespread use of highly effective cystic fibrosis transmembrane conductance regulator (CFTR) modulators, including the risk for undernutrition [6]. However, advancements in CF care have also led to significantly increased rates of overweight and obesity [13,14,15,16,17]. Additionally, the prevalence of non-pulmonary complications such as CF-related diabetes (CFRD) continues to increase, particularly as the CF population ages [18,19]. At present, there are few published studies investigating the impact of these high-calorie, lower-quality diets on body composition and the development of CF-related metabolic comorbidities [9,20].

While BMI has classically been the primary measure of nutritional outcomes in patients with CF, there is interest in evaluating other potentially more meaningful predictors of health status [9,20]. There is growing evidence that BMI may not accurately reflect body composition, particularly the distinction between fat mass and fat-free mass [21,22,23]. As a result, the use of BMI as the primary marker of nutritional status in CF may have significant drawbacks in routine clinical care.

Dual-energy X-ray absorptiometry (DXA) body composition analysis has become increasingly utilized in individuals with CF, particularly given its ability to provide more detailed information regarding the distribution of fat mass, lean mass, and bone density [20]. Several groups have described an increased prevalence of normal weight obesity (NWO) and decreased fat-free mass distribution (FFMD) in individuals with CF, as well as a link between this type of body habitus and poorer lung function [20,24,25]. There may exist specific DXA body composition variables that better predict long-term CF-specific clinical outcomes than BMI [26]. If identified, these variables could predict the risk of pulmonary decline and metabolic abnormalities in patients with CF, and help guide individualized advice regarding dietary composition and physical activity.

We performed a cross-sectional analysis comparing the dietary intake, physical activity, and DXA body composition measures in adolescents and adults with CF and age-, race- and gender-matched healthy volunteers. We also investigated how body composition correlated with pulmonary status and dietary intake in participants with CF. We hypothesized that adults with CF would have higher carbohydrate intake, greater fat mass, and lower lean mass than healthy volunteers, and that measures of lean mass would correlate more strongly with percent predicted forced expiratory volume in 1 second (FEV1) than BMI.

## 2. Materials and Methods

### 2.1. Participants and Eligibility Criteria

Cross-sectional data were analyzed from the baseline visit of a prospective observational study investigating the effect of ivacaftor on bone density and microarchitecture in individuals with CF [27]. Participants with CF were recruited from the Massachusetts General Hospital and Boston Children’s Hospital Cystic Fibrosis Center. Exclusion criteria for participants with CF included history of solid organ transplantation, current pregnancy, and *Burkholderia dolosa* infection (due to institutional infection control issues). Matched healthy volunteers were recruited from the community. Exclusion criteria for healthy volunteers included current pregnancy, a history of medications or disorders known to affect bone metabolism, cumulative use of oral glucocorticoids for greater than two months, or BMI < 18.5 or >30 kg/m^2^ (or <5th percentile or >95th percentile for pediatric participants) at the time of screening.

The parent study included children and adults with CF and at least one copy of the G551D mutation, who were matched by age (±2 years and by Tanner stage in pediatric participants), race, and gender to a cohort of participants with CF and other CFTR mutations, and to a cohort of healthy volunteers. For the present analysis, only post-pubertal (Tanner stage V) participants aged 15 years and above were included, due to the rapid and variable changes in diet and body composition occurring during growth. The protocol was approved by the Mass General Brigham Institutional Review Board (IRB) with ceded review by the Boston Children’s Hospital IRB and was registered on clinicaltrials.gov (NCT01549314). Written informed consent was obtained from all participants.

### 2.2. Clinical Assessments

All participants were queried regarding medical history, medication use including oral and inhaled glucocorticoids, alcohol and tobacco use, and pubertal and reproductive history. Tanner staging in pediatric participants was performed by a board-certified pediatric endocrinologist. Additional data obtained from participants with CF included the number of CF exacerbations in the past year, defined as treatment with intravenous antibiotics and/or hospitalization. CFTR genotype and percent predicted forced expiratory volume in 1 s (FEV1) in the most recent pulmonary function testing were obtained in participants with CF by chart review. In all participants, height was measured on a wall-mounted stadiometer and weight on an electronic scale. Race and ethnicity were self-reported. Serum glucose and insulin levels were obtained after fasting at least 8 h overnight; these data were used to calculate a homeostatic model assessment for insulin resistance (HOMA-IR), with a level >2 considered consistent with insulin resistance [28].

### 2.3. Dietary Intake and Physical Activity Assessments

A registered dietician assessed nutritional intake with a 24-h dietary recall. Dietary composition of fat (g), protein (g), and carbohydrates (g), percentage of calories from each macronutrient, total energy (kcal), added sugar (g), glycemic index (GI) and glycemic load as defined by the International Carbohydrate Quality Consortium (ICQC) [29], were quantified using the validated Nutrient Data System for Research (NDSR) [30]. Physical activity was also assessed by a registered dietician using the Modifiable Activity Questionnaire, a self-reported tool that assesses each individual’s degree of physical activity over the last 1 year, based on 40 leisure and occupational activity items, ranging in intensity [31].

### 2.4. Assessment of Body Composition

Whole and regional body composition analyses were obtained from whole body DXA scans (Discovery A, Hologic Inc., Bedford, MA, USA). To account for variants in stature, height-normalized indexes were determined for fat-mass and lean mass (mass in kg/height in m^2^). DXA quality control included daily measurement of a Hologic DXA anthropomorphic spine phantom and visual review of all images by an experienced investigator.

Participants were classified as normal weight (BMI 18–24.9 kg/m^2^), overweight (BMI 25–29.9 kg/m^2^), or obese (BMI ≥ 30 kg/m^2^). Normal weight obesity (NWO) was defined as those with a normal BMI < 25 kg/m^2^ but with a body fat percentage of >30% in women and >23% in men [20,32].

### 2.5. Statistical Analyses

Statistical analyses were performed using STATA (version 16, StataCorp LLC, College Station, TX, USA). Normality was assessed for all variables using the Shapiro–Wilk test. Clinical characteristics, dietary intake, and body composition measures were compared between participants with CF and healthy volunteers using independent *t*-tests or Wilcoxon rank sum tests for normally and non-normally distributed data, respectively. Categorical variables were compared using chi square tests. In participants with CF, the relationship between body composition measures and FEV1 or dietary intake variables was determined via Pearson or Spearman correlation analysis, for normally and non-normally distributed data, respectively. Multivariable regression analysis was used to assess the relationship between various body composition measures and FEV1 Two regression models were used to adjust for potential confounding effects: Model 1, adjusting for age and gender; Model 2, adjusting for age, gender and BMI. A *p*-value of <0.05 was considered statistically significant.

## 3. Results

### 3.1. Clinical Characteristics

Thirty-eight adolescents and adults with CF and 19 healthy volunteers were included in the analysis. Clinical characteristics are summarized in Table 1. Participants ranged in age from 15 to 56 years and included eight adolescents with CF and four adolescent healthy volunteers aged 15–17 years. There were no significant differences between age, gender, race/ethnicity, anthropometric measures, HOMA-IR, or physical activity between the participants with CF and the healthy volunteers. Of the participants with CF, 30 (78.9%) had a history of pancreatic insufficiency and 5 (13.2%) had a history of CFRD. Five of the participants with CF without CFRD had fasting glucose levels consistent with impaired fasting glucose (100–125 mg/dL). Approximately half of these individuals (*n* = 20, 52.6%) were heterozygous and seven (18.4%) were homozygous for the F508del mutation. As the initial CF cohort was recruited to study the effects of ivacaftor on BMD, half (*n* = 19) of these participants had the G551D mutation. Ten participants (26.3%), all of whom had the G551D mutation, were taking ivacaftor, which was initiated within three months of the study visit. No other modulators were available for clinical use at the time of study enrollment. In the 12 months prior to the study visit, thirteen (34%) of the participants with CF experienced one or more CF exacerbations and 15 (40%) reported treatment with systemic glucocorticoids. Three of the participants with CF were overweight (BMI ≥ 25 kg/m^2^), and two were obese (BMI ≥ 30 kg/m^2^). Of the remaining 32 participants, approximately one-third (*n* = 10, 31.25%) met criteria for NWO, including five women (33.3%) and five men (29.4%).

### 3.2. Body Composition and Dietary Intake in Participants with CF and Healthy Volunteers

Comparisons of DXA body composition measures between participants with CF and healthy volunteers are presented in Table 2. Participants with CF had a lower appendicular lean mass/height^2^ (appendicular lean mass index, ALMI) compared with the healthy volunteers. All other body composition measures were similar between the two cohorts. As shown in Table 2, participants with CF reported a significantly higher total fat intake, greater % calories from fat, higher glycemic index, and lower % calories from protein than the healthy volunteers.

### 3.3. Relationship between Body Composition and Pulmonary Function in Participants with CF

**Figure 1:**
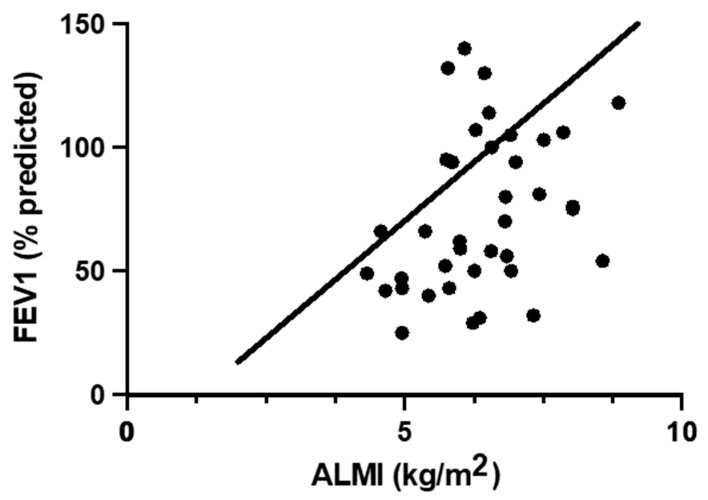
Multivariable regression of FEV1 vs. ALMI in participants with CF. Figure 1 displays the relationship between individuals’ FEV1 and ALMI values (circles) as well as the regression line of best fit across the whole dataset when controlling for age, gender and BMI (Model 2). FEV1, % predicted forced expiratory volume in 1 s; ALMI, appendicular lean mass index.

Table 3 presents the results of correlation analyses and multiple linear regression models for FEV1 and DXA body composition measures in the participants with CF (*n* = 38). On univariable correlation analysis, only ALMI was significantly correlated with FEV1. When controlling for age and gender (regression Model 1), FEV1 showed significant positive correlations with lean mass, lean mass/height^2^ (lean mass index, LMI), and ALMI. Subsequent analysis adjusting for age, gender, and BMI (regression Model 2) displayed an even stronger relationship between FEV1 and ALMI (Figure 1), but the relationship with LMI was no longer significant. In addition, a significant negative correlation between FEV1 and fat measures (% fat, trunk % fat, and fat mass/height^2^ (fat mass index, FMI)) was noted when age, gender, and BMI were included in the model. BMI was significantly correlated with FEV1; however, this significance was lost after adjusting for age and gender.

### 3.4. Relationship of Body Composition with Dietary Intake and Physical Activity in Participants with CF

Table 4 outlines the results of the correlation analysis between DXA body composition measures, dietary intake components, and physical activity scores. Multiple measures of body fat composition, particularly fat mass, % fat, and FMI, negatively correlated with total amount of macronutrients (energy, fat, carbohydrate and protein), though the relative proportion of each macronutrient intake did not correlate with body composition. Added sugar and glycemic load both negatively correlated with multiple measures of body fat, including % fat and FMI. Lean mass, LMI, and ALMI were not significantly associated with dietary composition. HOMA-IR did not significantly correlate with any DXA body composition measures or dietary variables (data not shown).

Physical activity score was negatively correlated with multiple measures of body fat, including fat mass, % fat, trunk fat mass, trunk % fat, and FMI. In contrast, ALMI was positively correlated with physical activity, with no other relationship noted between physical activity and other lean mass measures.

## 4. Discussion

In this cross-sectional study, individuals with CF had significantly lower ALMI compared with the healthy volunteers, despite no observed differences in BMI. In participants with CF, pulmonary function was positively associated with measures of lean mass but negatively associated with measures of fat mass when accounting for age, gender, and BMI, with ALMI having the strongest correlation. Higher physical activity levels were also correlated with greater ALMI and lower body fat measures. Participants with CF consumed significantly more fat, had higher glycemic index diets, and had a lower proportion of calories from protein than their healthy peers. Interestingly, measures of lean mass were not associated with key dietary intake variables in participants with CF; however, those with the lowest body fat had the greatest caloric intake, without a significant relationship to the relative macronutrient composition of their diet. 

BMI has been the primary measure of nutritional status in patients with CF for many years, due to its established strong correlation with pulmonary function and mortality [1,3,6]. However, BMI does not distinguish between fat mass and lean mass, and may be an insensitive marker of both fat-free mass deficits and excess adiposity in patients with CF [20,23,24]. For example, in a cross-sectional study of 86 adults with CF, fat-free mass depletion was found in 14% of participants, but was undetected by BMI in 58% of cases [23]. A study by Alvarez et al., of 32 adults with CF reported that 31% had NWO, defined as % fat >30% in women and >23% in men in the setting of a normal BMI, and that these subjects had a lower fat free mass index and pulmonary function than overweight subjects, suggesting that excess adiposity may impact clinical outcomes even in the setting of a normal BMI [20]. In our study, we found a similar proportion of participants with NWO (31%). In addition, we found no difference in BMI between participants with CF and healthy volunteers; however, those with CF had a significantly lower ALMI, which has been identified as an important marker of sarcopenia and low muscle mass [33]. Lower ALMI has been associated with increased mortality in the healthy older population [33], but this measure has not previously been reported in CF.

ALMI was also significantly correlated with pulmonary function in participants with CF in our study, with an even stronger relationship after adjusting for age, gender, and BMI. In contrast, BMI was correlated with FEV1 in the univariate analysis, but lost significance after adjustment for age and gender, suggesting that ALMI may be a superior measure than BMI in predicting lung function. After multivariable adjustment, lean mass was also positively associated with FEV1, whereas measures of body fat (fat mass, % fat, and FMI) were negatively correlated with FEV1. Similar to our findings, other studies in the CF population have reported associations of lower fat-free mass or lean body mass with lower pulmonary function [20,21,24]. Although ALMI correlations with clinical outcomes have not previously been reported in CF, appendicular lean mass (ALM) was found to be associated with FEV1 in one study of 69 adolescents with CF [5], and another study reported a greater number of CF exacerbations in those with lower appendicular fat-free mass [34]. In addition, our findings of a negative correlation between body fat measures and pulmonary function are consistent with the previously cited study by Alvarez et al., in which FMI was negatively associated with FEV1 after adjusting for age, gender and BMI [20]. Altogether, these results build a strong case supporting the importance of lean mass, particularly ALMI, in promoting lung function while implicating excess adiposity in pulmonary decline.

To achieve and maintain an adequate BMI, patients with CF are encouraged to consume a caloric intake of 120–150% of the dietary reference intake (DRI) for the typical healthy adult [1,35,36,37,38]. The CF Foundation, American Diabetes Association (ADA), and European Society for Clinical Nutrition and Metabolism (ESPEN) recommend a similar caloric composition for children and adults with CF, comprised of 20% of calories from protein, 35–50% from fat, and 40–50% from carbohydrate, though these recommendations are built on a general consensus rather than evidence-based data [1,36]. Current guidelines do not specify the composition of carbohydrate intake apart from avoiding artificial sweeteners and closely monitoring carbohydrate intake to maintain glycemic control [8,36,37,38].

Nutritional interventions in patients with CF often target increasing or maintaining BMI with high-calorie, high-carbohydrate and high-fat diets. However, in contrast with the trends observed in the general population, the impact of such diets on body composition in adults with CF is less clear [10,20]. In our study, we found that participants with CF consumed a higher calorie diet driven predominately by the intake of fat, and higher glycemic foods with a lower proportion of calories from protein, as compared with healthy volunteers. Similar to our findings, a cross-sectional study of 80 children with CF aged 2–18 years (mean age 9.3 years), with age- and gender-matched controls, found that children with CF consumed significantly more energy-dense, nutrient-poor foods than the controls. In addition, another study noted significantly higher added sugar intake, lower Healthy Eating Index scores, and higher visceral adipose tissue (VAT) in 24 adults with CF compared with the matched controls [9]. Interestingly, VAT was associated with higher added sugar intake and fasting glucose levels in that study. In contrast, we found a negative correlation between body fat measures and both added sugar and glycemic load, and a negative correlation was also noted with total energy intake as well as the absolute value of all macronutrients, irrespective of macronutrient intake as a proportion of total calories. No correlations were noted between diet and any measures of lean mass. Although unexpected, these results suggest that the relative macronutrient composition of the diet may not directly impact body composition, and that those patients with the lowest body fat were consuming the greatest number of calories from all sources, perhaps related to increased metabolic needs. 

Not surprisingly, physical activity levels were positively correlated with ALMI and negatively correlated with multiple measures of body fat, supporting the beneficial role of exercise on body composition and muscle health. Several prior studies have shown a strong correlation between fat-free mass (FFM) content and exercise capacity [39,40,41]. In a prospective pilot observational study of 28 adults with CF participating in an 8-week exercise training (ET) program, with 15 CF controls with no ET, Prevotat et al., found that ET resulted in an increased FFM compared with the controls [39]. Another study in 18 adolescents with CF reported that FFM measured by bioimpedance correlated with FVC z-score, maximal inspiratory pressure, and exercise tolerance. Interestingly, BMI did not significantly correlate with pulmonary or respiratory muscle function in this study.

Strengths of this study included the comprehensive clinical, DXA, and dietary measures prospectively collected in a relatively large number of patients with CF, as well as the inclusion of an age-, race-, and gender-matched healthy control group. However, important limitations of this study should be noted. The cross-sectional study design limited the conclusions on causality that could be drawn, and further prospective longitudinal studies investigating body composition in CF are needed. Participants were recruited from a single study center, potentially limiting the heterogeneity of the study population. Nutrition data were limited to a single 24-h diet recall, as opposed to 3-day food diary; repeated prospective data collection within participants may have provided more accurate dietary information. Similarly, physical activity was measured by survey and not by a wearable activity tracker or fitness monitor. Although there were multiple definitions of NWO in the literature, we utilized a definition that has previously been studied in CF. Given that the primary outcome for the parent study was to assess the effect of ivacaftor therapy on bone density and microarchitecture, half of the participants had the G551D mutation. A genotypically more diverse CF population may have impacted our outcomes. In addition, the small number of adolescent participants limited our ability to investigate differences between adolescents and adults. Lastly, few of the participants were on CFTR modulators at the time of the study, which could limit the applicability of these results to patients on highly effective CFTR modulator therapy.

## 5. Conclusions

In conclusion, body composition measures may more accurately predict key clinical outcomes in individuals with CF than BMI. In particular, ALMI was significantly lower in individuals with CF than healthy volunteers, despite no differences in BMI, which may have important clinical implications given the observed correlation between ALMI and pulmonary function. In contrast, FMI and other measures of body fat were negatively correlated with FEV1 when accounting for age, gender, and BMI, suggesting a detrimental effect of adiposity in CF. Our data also support the beneficial impact of physical activity on body composition, including increased ALMI and decreased body fat measures. Future prospective longitudinal studies analyzing the effect of body composition, dietary intake, and physical activity on CF-specific clinical outcomes are greatly needed, particularly in the post-modulator era.

## Figures and Tables

**Table 1:** Clinical Characteristics.

	CF (*n* = 38)	Healthy Volunteers (*n* = 19)	*p*-Value
Age (years)	27.9 ± 2.0	28.8 ± 2.7	0.796
Female, *n* (%)	20 (52.6%)	10 (52.6%)	
Race, *n* (%)			
White	38 (100%)	19 (100%)	
Black			
Asian			
Native Haqaiian or Pacific Islander			
American Indian or Alaskan Native			
Ethnicity, *n* (%)			
Hispanic	0 (0%)	0 (0%)	
Non-Hispanic	38 (100%)	38 (100%)	
Height (cm)	166.9 ± 1.5	170.3 ± 1.9	0.185
Weight (kg)	59.6 ± 1.9	64.4 ± 2.6	0.154
BMI (kg/m^2^)	21.4 ± 0.6	22.1 ± 0.5	0.447
HOMA-IR	1.2 ± 0.1	1.0 ± 0.1	0.455
Physical Activity Score	22.3 ± 2.8	16.4 ± 2.6	0.311
Genotype, *n* (%)			
F508del homozygous	7 (18.4%)		
F508del heterozygous	20 (52.6%)		
Other	11 (28.9%)		
Pancreatic insufficiency, *n* (%)	30 (78.9%)		
FEV1 (% predicted)	73 ± 5		
CFRD, *n* (%)	5 (13.2%)		
CF Exacerbations in the past prior year (Y/N)	25 (65.8%)		
Number of CF Exacerbations in the prior year	1.5 ± 0.25		
Glucocorticoid Use in the prior year	15 (39.5%)		
Ivacaftor Use, *n* (%)	10 (26.3%)		

Data displayed as mean ± standard error (SE) or *n* (%) unless otherwise indicated. BMI, body mass index kg/m^2^; FEV1, forced expiratory volume; CFRD, cystic fibrosis related diabetes; HOMA-IR, homeostatic model assessment for insulin resistance.

**Table 2:** Body composition and dietary intake in participants with CF and healthy volunteers.

	CF (*n* = 38)	Healthy Volunteers (*n* = 19)	*p*-Value
Body Composition			
Fat mass (g)	15,740 ± 1119	16,687 ± 898	0.097
Lean mass (g)	42,438 ± 1301	46,109 ± 2593	0.352
Total mass (g)	60,274 ± 1940	65,163 ± 2611	0.07
% fat	25.6 ± 1.2	26.2 ± 1.7	0.778
Trunk fat mass (g)	7051 ± 646	6709 ± 426	0.294
Trunk total mass (g)	30,267 ± 1111	30,330 ± 1299	0.618
Trunk % fat	22.3 ± 1.3	22.4 ± 1.3	0.52
Fat mass/height^2^ (kg/m^2^)	5.8 ± 0.4	5.9 ± 0.4	0.275
Lean/height^2^ (kg/m^2^)	15.9 ± 0.4	16.4 ± 0.6	0.481
Appen lean/height^2^ (kg/m^2^)	6.4 ± 0.2	7.2 ± 0.4	0.029
Dietary intake			
Energy (kcal)	2880 ± 258	2203 ± 196	0.137
Total Fat (g)	118.8 ± 11.7	74.8 ± 8	0.016
Total Carb (g)	362.6 ± 34.4	292 ± 22.3	0.418
Total Protein (g)	104 ± 10.2	96.5 ± 11.4	0.667
Cholesterol (mg)	313.9 ± 42.9	248.3 ± 26.9	0.569
% Calories from Fat	36.5 ± 1.6	29.3 ± 1.3	0.002
% Calories from Carbohydrates	48.5 ± 1.8	52.8 ± 1.4	0.092
% Calories from Protein	14.9 ± 0.8	17.2 ± 0.8	0.015
Added Sugar (g)	120.6 ± 17.5	66.7 ± 6.2	0.142
Glycemic Index	61.4 ± 1.2	57.7 ± 1.1	0.036
Glycemic Load	213.1 ± 22	154.1 ± 11.6	0.131

Data displayed as mean ±SE; CF, cystic fibrosis.

**Table 3:** Correlation analyses and multiple linear regression of FEV1 vs. body composition measures in participants with CF.

	% Predicted FEV1					
	r	*p*-Value	Model 1 Beta Coefficient	*p*-Value	Model 2 Beta Coefficient	*p*-Value
Fat mass (g)	0.174	0.296	0.0004 ± 0.0009	0.656	−0.003 ± 0.002	0.051
Lean mass (g)	0.194	0.243	**0.003 ± 0.001**	**0.001**	**0.003 ± 0.001**	**0.003**
% fat	0.065	0.7	−1.12 ± 0.992	0.263	**−4.066 ± 0.1.121**	**0.001**
Trunk fat mass (g)	0.157	0.348	0.001 ± 0.002	0.513	−0.005 ± 0.003	0.111
Trunk total mass (g)	0.265	0.108	0.002 ± 0.001	0.072	0.001 ± 0.002	0.451
Trunk % fat	0.081	0.629	−0.259 ± 0.87	0.768	**−3.206 ± 1.184**	**0.011**
FMI (kg/m^2^)	0.171	0.305	−0.141 ± 2.35	0.953	**−14.684 ± 3.931**	**0.001**
LMI (kg/m^2^)	0.264	0.11	**5.242 ± 2.325**	**0.031**	4.839 ± 3.459	0.171
ALMI (kg/m^2^)	**0.332**	**0.042**	**15.021 ± 4.45**	**0.002**	**18.972 ± 6.542**	**0.007**
BMI (kg/m^2^)	**0.405**	**0.012**	2.574 ± 1.505	0.096		

Data displayed as correlation coefficient/Spearman’s rho for correlation analyses or Beta coefficient ± SE for regression models. Model 1: adjusted for age and gender; model 2: adjusted for age, gender and BMI. Significant results (*p* < 0.05) are in bold. FEV1, % predicted forced expiratory volume in 1 s; ALMI, appendicular lean mass index; LMI, lean mass index; FMI, fat mass index.

**Table 4:** Correlation analyses between dietary intake and body composition in participants with CF.

	Energy (kcal)	Total Fat (g)	Total Carb (g)	Total Protein (g)	% Cal Fat	% Cal Carb	% Cal Protein	Added Sugars (g)	GI (Glucose)	GL (Glucose)	Physical Activity
Fat mass (g)	**−0.533 (0.002)**	**−0.498 (0.004)**	**−0.445 (0.012)**	**−0.508 (0.004)**	0.091 (0.626)	−0.104 (0.576)	0.096 (0.608)	−0.354 (0.051)	−0.036 (0.846)	**−0.445 (0.012)**	**−0.469 (0.003)**
Lean mass (g)	0.181 (0.331)	0.194 (0.296)	0.073 (0.696)	0.277 (0.131)	0.248 (0.179)	−0.26 (0.158)	0.161 (0.389)	0.005 (0.979)	−0.024 (0.9)	0.072 (0.70)	0.304 (0.068)
% fat	**−0.679 (<0.0001)**	**−0.621 (0.0002)**	**−0.512 (0.003)**	**−0.705 (<0.0001)**	−0.041 (0.827)	0.084 (0.653)	0.015 (0.936)	**−0.409 (0.022)**	−0.024 (0.898)	**−0.481 (0.006)**	**−0.55 (0.0004)**
Trunk fat mass (g)	**−0.54 (0.002)**	**−0.504 (0.004)**	**−0.443 (0.013)**	**−0.536 (0.002)**	0.084 (0.655)	−0.08 (0.668)	0.017 (0.928)	−0.334 (0.066)	−0.057 (0.763)	**−0.451 (0.011)**	**−0.457 (0.005)**
Trunk % fat	**−0.65 (0.0001)**	**−0.618 (0.0002)**	**−0.512 (0.003)**	**−0.684 (<0.0001)**	0.016 (0.931)	−0.009 (0.962)	−0.02 (0.916)	−0.349 (0.054)	−0.035 (0.853)	**−0.508 (0.004)**	**−0.532 (0.0007)**
FMI (kg/m^2^)	**−0.626 (0.0002)**	**−0.567 (0.0009)**	**−0.525 (0.002)**	**−0.623 (0.0002)**	0.097 (0.605)	−0.097 (0.604)	0.049 (0.794)	**−0.409 (0.022)**	−0.051 (0.784)	**−0.517 (0.003)**	**−0.51 (0.001)**
LMI (kg/m^2^)	−0.135 (0.469)	−0.099 (0.595)	−0.223 (0.228)	0.012 (0.949)	0.192 (0.301)	−0.271 (0.14)	0.259 (0.159)	−0.196 (0.292)	−0.156 (0.403)	−0.228 (0.219)	0.225 (0.181)
ALMI (kg/m^2^)	0.004 (0.984)	0.063 (0.737)	−0.029 (0.878)	0.153 (0.41)	0.206 (0.266)	−0.288 (0.116)	0.232 (0.209)	−0.062 (0.742)	−0.113 (0.546)	−0.043 (0.819)	**0.367 (0.025)**

Data displayed as correlation coefficient/Spearman’s rho (*p*-value). Significant results (*p* < 0.05) are in bold. CF, cystic fibrosis, ALMI, appendicular lean mass index; LMI, lean mass index; FMI, fat mass index.

## Data Availability

Some or all datasets generated during and/or analyzed during the current study are not publicly available but are available from the corresponding author on reasonable request.
